# Benzene-1,3,5-triyl triacetate

**DOI:** 10.1107/S1600536809050016

**Published:** 2009-11-28

**Authors:** Alan H. Haines, David L. Hughes

**Affiliations:** a School of Chemistry, University of East Anglia, Norwich NR4 7TJ, England

## Abstract

The asymmetric unit of the title compound, C_12_H_12_O_6_, contains two essentially identical mol­ecules related by a pseudo-inversion centre. The three acet­oxy groups in each mol­ecule are essentially planar and are tilted, in a regular propeller-style arrangement, with their normals oriented between 56.72 (12) and 76.35 (9)° from the normal to the mean plane of the central C_6_ ring; in each mol­ecule the three carbonyl O atoms are on the same side of the C_6_ ring, with the C_ring_—O—C—Me bonds in a *trans* conformation. The principal inter­molecular contacts appear to be C—H⋯π-ring inter­actions; each C_6_ ring has such a contact to both faces of the ring; in addition, each mol­ecule has two inter­molecular C—H⋯O contacts with H⋯O distances less than 2.55 Å.

## Related literature

For our previous studies in this area, see: Haines & Hughes (2007 ▶); Haines *et al.* (2008 ▶, 2009 ▶). For a related structure, see: Haines & Hughes (2009 ▶). 
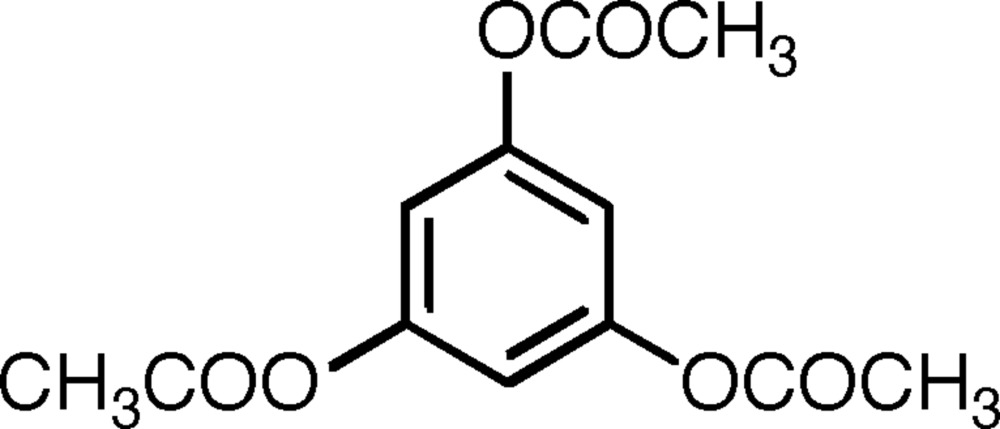
/p>

## Experimental

### 

#### Crystal data

C_12_H_12_O_6_
*M* *_r_* = 252.22
Monoclinic, 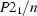

*a* = 6.20290 (16) Å
*b* = 24.6643 (6) Å
*c* = 15.3862 (4) Å
β = 95.297 (2)°
*V* = 2343.88 (11) Å^3^
*Z* = 8
Mo- *K*α radiation
μ = 0.12 mm^−1^
*T* = 140 K
0.38 × 0.18 × 0.17 mm

#### Data collection

Oxford Diffraction Xcalibur 3/CCD diffractometer
Absorption correction: multi-scan (*CrysAlisPro RED*; Oxford Diffraction, 2008 ▶) *T*_min_ = 0.931, *T*_max_ = 1.041
40881 measured reflections
4128 independent reflections
2769 reflections with *I* > 2σ(*I*)
*R*_int_ = 0.054

#### Refinement

*R*[*F* ^2^ > 2σ(*F* ^2^)] = 0.065
*wR*(*F* ^2^) = 0.196
*S* = 1.08
4128 reflections
331 parameters
H-atom parameters constrained
Δρ_max_ = 0.65 e Å^−3^
Δρ_min_ = −0.26 e Å^−3^

### 

Data collection: *CrysAlisPro CCD* (Oxford Diffraction, 2008 ▶); cell refinement: *CrysAlisPro RED* (Oxford Diffraction, 2008 ▶); data reduction: *CrysAlisPro RED*; program(s) used to solve structure: *SHELXS97* (Sheldrick, 2008 ▶); program(s) used to refine structure: *SHELXL97* (Sheldrick, 2008 ▶); molecular graphics: *ORTEP-3* (Farrugia, 1997 ▶); software used to prepare material for publication: *SHELXL97*.

## Supplementary Material

Crystal structure: contains datablocks I, global. DOI: 10.1107/S1600536809050016/hb5176sup1.cif

Structure factors: contains datablocks I. DOI: 10.1107/S1600536809050016/hb5176Isup2.hkl

Additional supplementary materials:  crystallographic information; 3D view; checkCIF report

## Figures and Tables

**Table 1:** Hydrogen-bond geometry (Å, °)

*D*—H⋯*A*	*D*—H	H⋯*A*	*D*⋯*A*	*D*—H⋯*A*
C52—H52*A*⋯O31^i^	0.96	2.53	3.363 (4)	145
C52—H52*C*⋯O11^i^	0.96	2.31	3.242 (4)	163
C932—H93*A*⋯O911^ii^	0.96	2.51	3.292 (4)	139
C952—H95*C*⋯O951^iii^	0.96	2.54	3.474 (4)	165
C4—H4⋯*Cg*2^iv^	0.93	2.68	3.460 (3)	141
C12—H12*C*⋯*Cg*1^v^	0.96	2.86	3.604 (4)	135
C912—H91*B*⋯*Cg*2^vi^	0.96	2.84	3.658 (4)	143
C94—H94⋯*Cg*1^vii^	0.93	2.67	3.435 (3)	140

Symmetry codes: (i) 
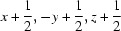
; (ii) 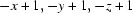; (iii) 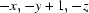; (iv) 
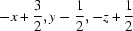
; (v) 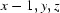; (vi) 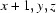; (vii) 
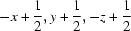
. *Cg*1 and *Cg*2 are the centroids of the C1–C6 and C91–C96 rings, respectively.

**Fig. 1.**
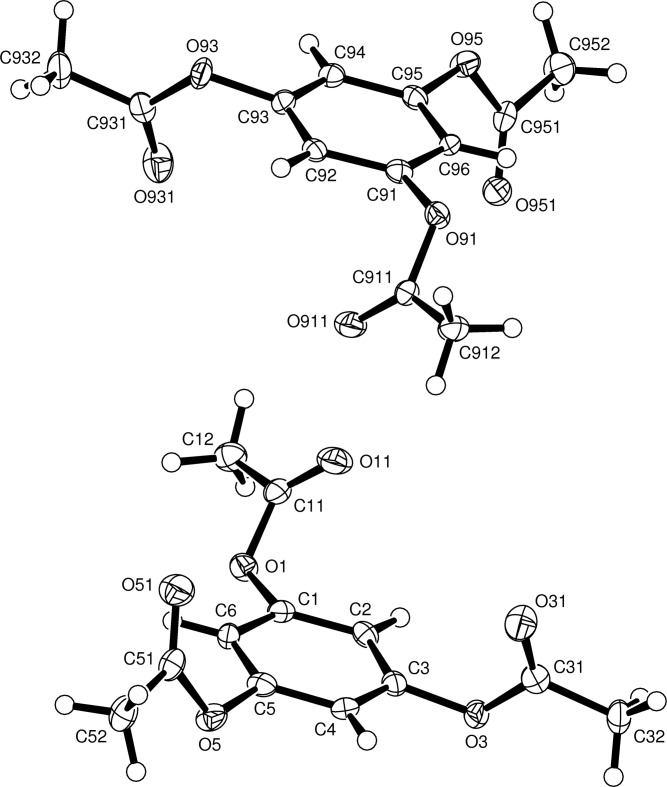
View of the two independent molecules in (I), related by a pseudo-inversion centre at ca 0, 1/4, 1/2. Displacement ellipsoids are drawn at the 50% probability level.

## supplementary crystallographic information

### Comment

Structural factors which enhance the solubility of organic compounds in liquid carbon dioxide are difficult to identify, but a knowledge of these is important in view of the possibility of using liquid carbon dioxide as an environmentally acceptable, cheap, safe and readily available alternative to replace organic-based solvents in the development of so-called "green chemistry". Previous studies (Haines *et al.*, 2008) have shown that certain types of acyl group promote the solubilities of per-acylated *D*-glucopyranose derivatives in liquid carbon dioxide; in particular trimethylacetyl groups promoted solubility, their effect being comparable to acetyl groups and superior to dimethylacetyl groups. In searching for an explanation for solubility differences in this series based on differing intermolecular forces in the solid state, we conducted crystal structure studies on the compounds (Haines & Hughes, 2007), but the results indicated no substantial difference in such intermolecular forces.

Measurement of solubilities in liquid carbon dioxide of the series of 1,3,5-triacetoxybenzene (1) and substituted derivatives, *viz *1,3,5-tris-(dimethylacetoxy)benzene (2) and 1,3,5-tris-(trimethylacetoxy)benzene (3), chosen in an attempt to separate the effects on solubility of the number and structure of peripheral substituents in compounds of similar overall molecular dimensions to the carbohydrate derivatives, showed no major differences (Haines, *et al.*, 2009, unpublished results) and prompted an investigation of their crystal structures in order to compare intermolecular interactions in these compounds.

The stucture of the first compound of the series, (1) is shown in Figure 1; other compounds of the series are described in the accompanying paper (Haines and Hughes, 2009). Dimensions are available in the archived CIFs.

Compound (1) was prepared by the acylation of 1,3,5-trihydroxybenzene with acetic anhydride and formed crystals having two essentially identical molecules with very similar orientations in the cell, related by a pseudo inversion centre. The three acetoxy groups in each molecule are essentially planar and are tilted, in a regular propeller-style arrangement, with their normals at 56.72 (12), 76.35 (9) and 60.73 (12)° from the normal to the mean-plane of the central C_6_ ring in one molecule and 58.44 (11), 75.11 (13) and 63.76 (11) ° in the second molecule. In each molecule the three carbonyl O-atoms are on the same side of the C_6_ ring, with the C_ring_—O—C—Me bonds in a *trans* conformation. The principal intermolecular contacts appear to be C—H···π-ring interactions: each C_6_ ring has such a contact to both faces of the ring – the ring C(1–6) has H(12C*^a^*) and H(94*^b^*) on opposite sides of the ring at 3.25 and 2.63 Å from the ring mean-plane, and the ring of C(91–96) is bounded by H(4*^c^*) and H(91B*^d^*) at 2.65 and 3.14 Å from the ring mean-plane (the superscripts *a*–*d* indicate symmetry operations). Also, each molecule makes two intermolecular C—H···O contacts with H···O distances in the range 2.31–2.54 Å.

### Experimental

The title compound was prepared by the conventional acylation of the parent 1,3,5-trihydroxybenzene and has been described previously (Hegetschweiler *et al.*, 1990); the physical data (m.p., ^1^H and ^13^C NMR spectra) of our product agreed with those reported.

To a solution of anhydrous 1,3,5-trihydroxybenzene (phloroglucinol) (1.26 g) in pyridine (6 ml) was added acetic anhydride (5.6 ml) and after 12 h the solution was poured into iced water which led to formation of a white precipitate. After stirring for 2 h, the solid was collected by filtration, and recrystallized from ethanol to give compound 1 (1.67 g, 66%), m.p. 106–107 °C (lit. {Hegetschweiler *et al.*, 1990} 106 °C); δ_H_(CDCl_3_) 6.84, (s, 3H), 2.27 (s, 9H); δ_C_(CDCl_3_) 168.65, 151.19, 112.76, 20.91. The NMR data are in full agreement with the reported literature values (Hegetschweiler *et al.*, 1990).

### Refinement

Hydrogen atoms were included in idealized positions and their *U*_iso_ values were set to ride on the *U*_eq_ values of the parent carbon atoms.

### Crystal data

**Table:**

C_12_H_12_O_6_	*Z* = 8
*M**_r_* = 252.22	*F*(000) = 1056
Monoclinic, *P*2_1_/*n*	*D*_x_ = 1.429 Mg m^−^^3^
Hall symbol: -P 2yn	Mo *K*α radiation, λ = 0.71073 Å
*a* = 6.20290 (16) Å	µ = 0.12 mm^−^^1^
*b* = 24.6643 (6) Å	*T* = 140 K
*c* = 15.3862 (4) Å	Prism, colourless
β = 95.297 (2)°	0.38 × 0.18 × 0.17 mm
*V* = 2343.88 (11) Å^3^	

### Data collection

**Table:**

Oxford Diffraction Xcalibur 3/CCD diffractometer	4128 independent reflections
Radiation source: Enhance (Mo) X-ray Source	2769 reflections with *I* > 2σ(*I*)
graphite	*R*_int_ = 0.054
Detector resolution: 16.0050 pixels mm^-1^	θ_max_ = 25.0°, θ_min_ = 3.1°
Thin–slice φ and ω scans	*h* = −7→7
Absorption correction: multi-scan (*CrysAlis PRO* RED; Oxford Diffraction, 2008)	*k* = −29→29
*T*_min_ = 0.931, *T*_max_ = 1.041	*l* = −18→18
40881 measured reflections	

### Refinement

**Table:**

Refinement on *F*^2^	Primary atom site location: structure-invariant direct methods
Least-squares matrix: full	Secondary atom site location: difference Fourier map
*R*[*F*^2^ > 2σ(*F*^2^)] = 0.065	Hydrogen site location: inferred from neighbouring sites
*wR*(*F*^2^) = 0.196	H-atom parameters constrained
*S* = 1.08	*w* = 1/[σ^2^(*F*_o_^2^) + (0.1157*P*)^2^ + 0.643*P*] where *P* = (*F*_o_^2^ + 2*F*_c_^2^)/3
4128 reflections	(Δ/σ)_max_ = 0.001
331 parameters	Δρ_max_ = 0.65 e Å^−^^3^
0 restraints	Δρ_min_ = −0.26 e Å^−^^3^

### Special details

**Table:**

Experimental. CrysAlisPro RED, Oxford Diffraction Ltd., Version 1.171.32.24 Empirical absorption correction using spherical harmonics, implemented in SCALE3 ABSPACK scaling algorithm.
Geometry. All e.s.d.'s (except the e.s.d. in the dihedral angle between two l.s. planes) are estimated using the full covariance matrix. The cell e.s.d.'s are taken into account individually in the estimation of e.s.d.'s in distances, angles and torsion angles; correlations between e.s.d.'s in cell parameters are only used when they are defined by crystal symmetry. An approximate (isotropic) treatment of cell e.s.d.'s is used for estimating e.s.d.'s involving l.s. planes.
Refinement. Refinement of *F*^2^ against ALL reflections. The weighted *R*-factor *wR* and goodness of fit *S* are based on *F*^2^, conventional *R*-factors *R* are based on *F*, with *F* set to zero for negative *F*^2^. The threshold expression of *F*^2^ > σ(*F*^2^) is used only for calculating *R*-factors(gt) *etc*. and is not relevant to the choice of reflections for refinement. *R*-factors based on *F*^2^ are statistically about twice as large as those based on *F*, and *R*- factors based on ALL data will be even larger.

### Fractional atomic coordinates and isotropic or equivalent isotropic displacement parameters (Å^2^)

**Table:**

	*x*	*y*	*z*	*U*_iso_*/*U*_eq_	
C1	0.1644 (5)	0.22484 (12)	0.2555 (2)	0.0171 (7)	
C2	0.2182 (5)	0.21091 (12)	0.17203 (19)	0.0171 (7)	
H2	0.1296	0.2200	0.1221	0.020*	
C3	0.4090 (5)	0.18306 (12)	0.16789 (19)	0.0188 (7)	
C4	0.5456 (5)	0.16979 (12)	0.24048 (19)	0.0177 (7)	
H4	0.6756	0.1518	0.2353	0.021*	
C5	0.4830 (5)	0.18408 (13)	0.32137 (19)	0.0191 (7)	
C6	0.2907 (5)	0.21159 (12)	0.33028 (19)	0.0176 (7)	
H6	0.2493	0.2207	0.3850	0.021*	
O1	−0.0344 (3)	0.24931 (9)	0.26764 (13)	0.0208 (5)	
C11	−0.0865 (5)	0.29755 (13)	0.2254 (2)	0.0216 (7)	
O11	0.0352 (4)	0.32021 (9)	0.18212 (16)	0.0330 (6)	
C12	−0.3065 (5)	0.31541 (14)	0.2446 (2)	0.0255 (8)	
H12A	−0.3271	0.3527	0.2281	0.038*	
H12B	−0.3206	0.3115	0.3059	0.038*	
H12C	−0.4137	0.2935	0.2122	0.038*	
O3	0.4578 (3)	0.16404 (8)	0.08570 (13)	0.0210 (5)	
C31	0.6135 (5)	0.19045 (13)	0.0455 (2)	0.0211 (7)	
O31	0.7100 (4)	0.22877 (10)	0.07680 (15)	0.0301 (6)	
C32	0.6401 (5)	0.16603 (14)	−0.04125 (19)	0.0255 (8)	
H32A	0.5192	0.1759	−0.0816	0.038*	
H32B	0.6469	0.1273	−0.0359	0.038*	
H32C	0.7714	0.1792	−0.0622	0.038*	
O5	0.6130 (3)	0.16564 (9)	0.39428 (13)	0.0233 (5)	
C51	0.7090 (5)	0.20210 (14)	0.4523 (2)	0.0222 (7)	
O51	0.6831 (4)	0.25004 (10)	0.44501 (15)	0.0325 (6)	
C52	0.8440 (5)	0.17336 (15)	0.5235 (2)	0.0265 (8)	
H52A	0.9872	0.1885	0.5292	0.040*	
H52B	0.8518	0.1355	0.5096	0.040*	
H52C	0.7797	0.1776	0.5775	0.040*	
C91	0.8316 (5)	0.52607 (12)	0.23650 (19)	0.0169 (7)	
C92	0.7795 (5)	0.53882 (12)	0.32051 (19)	0.0155 (7)	
H92	0.8699	0.5293	0.3697	0.019*	
C93	0.5880 (5)	0.56608 (12)	0.32707 (19)	0.0177 (7)	
C94	0.4486 (5)	0.58031 (12)	0.25507 (19)	0.0181 (7)	
H94	0.3188	0.5981	0.2614	0.022*	
C95	0.5094 (5)	0.56707 (12)	0.17385 (18)	0.0170 (7)	
C96	0.7021 (5)	0.54049 (11)	0.16326 (19)	0.0162 (7)	
H96	0.7423	0.5327	0.1079	0.019*	
O91	1.0296 (3)	0.50177 (9)	0.22335 (13)	0.0189 (5)	
C911	1.0768 (5)	0.45224 (13)	0.26152 (19)	0.0184 (7)	
O911	0.9526 (4)	0.42894 (9)	0.30330 (16)	0.0317 (6)	
C912	1.2938 (6)	0.43354 (14)	0.2422 (2)	0.0240 (7)	
H91A	1.3171	0.3972	0.2635	0.036*	
H91B	1.4021	0.4571	0.2703	0.036*	
H91C	1.3030	0.4342	0.1803	0.036*	
O93	0.5437 (3)	0.58395 (9)	0.41005 (13)	0.0224 (5)	
C931	0.3854 (5)	0.55818 (14)	0.4494 (2)	0.0211 (7)	
O931	0.2877 (4)	0.52004 (11)	0.41682 (15)	0.0315 (6)	
C932	0.3602 (6)	0.58228 (15)	0.5361 (2)	0.0290 (9)	
H93A	0.2355	0.5669	0.5594	0.043*	
H93B	0.3420	0.6208	0.5302	0.043*	
H93C	0.4870	0.5747	0.5749	0.043*	
O95	0.3780 (3)	0.58607 (9)	0.10101 (13)	0.0217 (5)	
C951	0.2842 (5)	0.54966 (13)	0.04289 (19)	0.0196 (7)	
O951	0.3003 (3)	0.50160 (9)	0.05213 (13)	0.0241 (5)	
C952	0.1636 (5)	0.57803 (15)	−0.0327 (2)	0.0273 (8)	
H95A	0.2478	0.5771	−0.0819	0.041*	
H95B	0.1380	0.6150	−0.0171	0.041*	
H95C	0.0277	0.5601	−0.0474	0.041*	

### Atomic displacement parameters (Å^2^)

**Table:**

	*U*^11^	*U*^22^	*U*^33^	*U*^12^	*U*^13^	*U*^23^
C1	0.0166 (16)	0.0130 (15)	0.0220 (16)	−0.0003 (13)	0.0030 (12)	0.0001 (12)
C2	0.0153 (16)	0.0180 (17)	0.0176 (15)	−0.0033 (14)	−0.0001 (12)	0.0030 (13)
C3	0.0249 (17)	0.0158 (16)	0.0158 (15)	−0.0062 (14)	0.0019 (13)	−0.0008 (12)
C4	0.0195 (16)	0.0127 (16)	0.0208 (16)	−0.0002 (13)	0.0012 (13)	−0.0017 (12)
C5	0.0215 (16)	0.0151 (16)	0.0195 (16)	−0.0029 (13)	−0.0047 (13)	0.0001 (12)
C6	0.0208 (17)	0.0176 (17)	0.0150 (15)	−0.0015 (14)	0.0051 (12)	−0.0013 (13)
O1	0.0186 (11)	0.0217 (12)	0.0225 (12)	0.0032 (9)	0.0034 (9)	0.0036 (9)
C11	0.0286 (18)	0.0173 (17)	0.0186 (16)	−0.0003 (14)	0.0005 (14)	−0.0033 (13)
O11	0.0367 (14)	0.0195 (13)	0.0459 (15)	0.0016 (11)	0.0193 (12)	0.0053 (11)
C12	0.0261 (17)	0.0234 (19)	0.0261 (18)	0.0035 (17)	−0.0022 (14)	−0.0001 (14)
O3	0.0263 (12)	0.0209 (12)	0.0159 (11)	−0.0037 (10)	0.0025 (9)	−0.0021 (9)
C31	0.0166 (16)	0.0240 (18)	0.0227 (17)	0.0027 (14)	0.0020 (13)	0.0025 (14)
O31	0.0279 (13)	0.0334 (14)	0.0294 (13)	−0.0088 (11)	0.0052 (10)	−0.0059 (11)
C32	0.0285 (18)	0.033 (2)	0.0159 (16)	0.0031 (15)	0.0049 (14)	0.0009 (14)
O5	0.0276 (12)	0.0208 (12)	0.0203 (11)	0.0033 (10)	−0.0051 (9)	−0.0036 (9)
C51	0.0174 (16)	0.0282 (19)	0.0214 (17)	0.0000 (14)	0.0050 (13)	−0.0055 (14)
O51	0.0434 (15)	0.0262 (14)	0.0262 (13)	−0.0040 (12)	−0.0056 (11)	−0.0018 (11)
C52	0.0247 (18)	0.036 (2)	0.0184 (16)	0.0039 (16)	−0.0025 (14)	−0.0049 (14)
C91	0.0150 (16)	0.0148 (15)	0.0210 (16)	−0.0036 (13)	0.0019 (13)	0.0006 (12)
C92	0.0179 (16)	0.0137 (16)	0.0144 (15)	−0.0037 (13)	−0.0010 (12)	0.0004 (12)
C93	0.0224 (16)	0.0153 (16)	0.0158 (15)	−0.0030 (13)	0.0031 (12)	−0.0027 (12)
C94	0.0200 (16)	0.0148 (16)	0.0199 (16)	−0.0029 (13)	0.0035 (13)	−0.0016 (12)
C95	0.0185 (15)	0.0162 (16)	0.0157 (15)	−0.0002 (13)	−0.0018 (12)	0.0031 (12)
C96	0.0241 (17)	0.0114 (16)	0.0139 (14)	−0.0019 (13)	0.0053 (12)	−0.0021 (12)
O91	0.0177 (11)	0.0205 (12)	0.0190 (11)	0.0034 (9)	0.0045 (9)	0.0034 (9)
C911	0.0241 (17)	0.0168 (16)	0.0142 (15)	−0.0007 (14)	0.0008 (13)	−0.0021 (12)
O911	0.0407 (14)	0.0178 (12)	0.0398 (14)	0.0016 (11)	0.0209 (12)	0.0034 (11)
C912	0.0276 (17)	0.0188 (18)	0.0255 (17)	0.0052 (16)	0.0015 (13)	0.0004 (14)
O93	0.0275 (12)	0.0262 (13)	0.0143 (11)	−0.0010 (10)	0.0061 (9)	−0.0055 (9)
C931	0.0170 (16)	0.0264 (19)	0.0203 (16)	0.0076 (14)	0.0039 (13)	0.0062 (14)
O931	0.0282 (13)	0.0416 (16)	0.0249 (13)	−0.0075 (12)	0.0036 (10)	−0.0011 (11)
C932	0.0313 (19)	0.039 (2)	0.0177 (17)	0.0069 (17)	0.0072 (14)	0.0040 (14)
O95	0.0251 (12)	0.0194 (12)	0.0192 (11)	0.0044 (9)	−0.0050 (9)	−0.0028 (9)
C951	0.0176 (16)	0.0255 (18)	0.0160 (16)	−0.0011 (14)	0.0040 (12)	−0.0044 (14)
O951	0.0297 (13)	0.0219 (13)	0.0205 (12)	−0.0041 (10)	0.0012 (9)	−0.0015 (9)
C952	0.0292 (19)	0.030 (2)	0.0210 (17)	0.0029 (16)	−0.0061 (14)	0.0004 (14)

### Geometric parameters (Å, °)

**Table:**

C1—C6	1.370 (4)	C91—C96	1.369 (4)
C1—C2	1.399 (4)	C91—C92	1.397 (4)
C1—O1	1.401 (3)	C91—O91	1.398 (3)
C2—C3	1.375 (4)	C92—C93	1.376 (4)
C2—H2	0.9300	C92—H92	0.9300
C3—C4	1.378 (4)	C93—C94	1.386 (4)
C3—O3	1.408 (4)	C93—O93	1.402 (3)
C4—C5	1.383 (4)	C94—C95	1.377 (4)
C4—H4	0.9300	C94—H94	0.9300
C5—C6	1.390 (4)	C95—C96	1.386 (4)
C5—O5	1.396 (3)	C95—O95	1.404 (3)
C6—H6	0.9300	C96—H96	0.9300
O1—C11	1.380 (4)	O91—C911	1.375 (4)
C11—O11	1.191 (4)	C911—O911	1.195 (4)
C11—C12	1.489 (4)	C911—C912	1.479 (4)
C12—H12A	0.9600	C912—H91A	0.9600
C12—H12B	0.9600	C912—H91B	0.9600
C12—H12C	0.9600	C912—H91C	0.9600
O3—C31	1.360 (4)	O93—C931	1.359 (4)
C31—O31	1.196 (4)	C931—O931	1.203 (4)
C31—C32	1.488 (4)	C931—C932	1.481 (4)
C32—H32A	0.9600	C932—H93A	0.9600
C32—H32B	0.9600	C932—H93B	0.9600
C32—H32C	0.9600	C932—H93C	0.9600
O5—C51	1.365 (4)	O95—C951	1.359 (4)
C51—O51	1.197 (4)	C951—O951	1.197 (4)
C51—C52	1.495 (4)	C951—C952	1.496 (4)
C52—H52A	0.9600	C952—H95A	0.9600
C52—H52B	0.9600	C952—H95B	0.9600
C52—H52C	0.9600	C952—H95C	0.9600
			
C6—C1—C2	123.1 (3)	C96—C91—C92	122.3 (3)
C6—C1—O1	115.7 (3)	C96—C91—O91	116.7 (3)
C2—C1—O1	121.0 (3)	C92—C91—O91	120.8 (3)
C3—C2—C1	116.4 (3)	C93—C92—C91	116.9 (3)
C3—C2—H2	121.8	C93—C92—H92	121.5
C1—C2—H2	121.8	C91—C92—H92	121.5
C2—C3—C4	123.3 (3)	C92—C93—C94	122.9 (3)
C2—C3—O3	117.7 (3)	C92—C93—O93	117.6 (3)
C4—C3—O3	118.8 (3)	C94—C93—O93	119.2 (3)
C3—C4—C5	117.8 (3)	C95—C94—C93	117.5 (3)
C3—C4—H4	121.1	C95—C94—H94	121.2
C5—C4—H4	121.1	C93—C94—H94	121.2
C4—C5—C6	121.9 (3)	C94—C95—C96	122.0 (3)
C4—C5—O5	116.8 (3)	C94—C95—O95	117.2 (3)
C6—C5—O5	121.1 (3)	C96—C95—O95	120.5 (3)
C1—C6—C5	117.6 (3)	C91—C96—C95	118.2 (3)
C1—C6—H6	121.2	C91—C96—H96	120.9
C5—C6—H6	121.2	C95—C96—H96	120.9
C11—O1—C1	118.7 (2)	C911—O91—C91	118.3 (2)
O11—C11—O1	122.3 (3)	O911—C911—O91	122.3 (3)
O11—C11—C12	127.7 (3)	O911—C911—C912	127.0 (3)
O1—C11—C12	109.9 (3)	O91—C911—C912	110.7 (3)
C11—C12—H12A	109.5	C911—C912—H91A	109.5
C11—C12—H12B	109.5	C911—C912—H91B	109.5
H12A—C12—H12B	109.5	H91A—C912—H91B	109.5
C11—C12—H12C	109.5	C911—C912—H91C	109.5
H12A—C12—H12C	109.5	H91A—C912—H91C	109.5
H12B—C12—H12C	109.5	H91B—C912—H91C	109.5
C31—O3—C3	118.0 (2)	C931—O93—C93	118.0 (2)
O31—C31—O3	123.1 (3)	O931—C931—O93	122.5 (3)
O31—C31—C32	126.0 (3)	O931—C931—C932	126.8 (3)
O3—C31—C32	110.9 (3)	O93—C931—C932	110.7 (3)
C31—C32—H32A	109.5	C931—C932—H93A	109.5
C31—C32—H32B	109.5	C931—C932—H93B	109.5
H32A—C32—H32B	109.5	H93A—C932—H93B	109.5
C31—C32—H32C	109.5	C931—C932—H93C	109.5
H32A—C32—H32C	109.5	H93A—C932—H93C	109.5
H32B—C32—H32C	109.5	H93B—C932—H93C	109.5
C51—O5—C5	119.7 (2)	C951—O95—C95	119.1 (2)
O51—C51—O5	122.9 (3)	O951—C951—O95	123.4 (3)
O51—C51—C52	126.8 (3)	O951—C951—C952	125.8 (3)
O5—C51—C52	110.4 (3)	O95—C951—C952	110.8 (3)
C51—C52—H52A	109.5	C951—C952—H95A	109.5
C51—C52—H52B	109.5	C951—C952—H95B	109.5
H52A—C52—H52B	109.5	H95A—C952—H95B	109.5
C51—C52—H52C	109.5	C951—C952—H95C	109.5
H52A—C52—H52C	109.5	H95A—C952—H95C	109.5
H52B—C52—H52C	109.5	H95B—C952—H95C	109.5
			
C6—C1—C2—C3	−0.6 (5)	C96—C91—C92—C93	1.1 (4)
O1—C1—C2—C3	−174.8 (3)	O91—C91—C92—C93	175.6 (3)
C1—C2—C3—C4	−1.1 (4)	C91—C92—C93—C94	0.7 (4)
C1—C2—C3—O3	174.2 (2)	C91—C92—C93—O93	−173.7 (2)
C2—C3—C4—C5	1.9 (5)	C92—C93—C94—C95	−1.2 (5)
O3—C3—C4—C5	−173.4 (3)	O93—C93—C94—C95	173.1 (3)
C3—C4—C5—C6	−0.9 (5)	C93—C94—C95—C96	0.0 (5)
C3—C4—C5—O5	173.9 (3)	C93—C94—C95—O95	−174.1 (3)
C2—C1—C6—C5	1.5 (5)	C92—C91—C96—C95	−2.2 (4)
O1—C1—C6—C5	175.9 (3)	O91—C91—C96—C95	−176.9 (3)
C4—C5—C6—C1	−0.7 (5)	C94—C95—C96—C91	1.6 (5)
O5—C5—C6—C1	−175.3 (3)	O95—C95—C96—C91	175.6 (3)
C6—C1—O1—C11	127.7 (3)	C96—C91—O91—C911	−125.3 (3)
C2—C1—O1—C11	−57.7 (4)	C92—C91—O91—C911	59.9 (4)
C1—O1—C11—O11	−3.2 (4)	C91—O91—C911—O911	1.9 (4)
C1—O1—C11—C12	178.0 (2)	C91—O91—C911—C912	−178.7 (2)
C2—C3—O3—C31	105.9 (3)	C92—C93—O93—C931	−108.7 (3)
C4—C3—O3—C31	−78.6 (3)	C94—C93—O93—C931	76.7 (4)
C3—O3—C31—O31	0.1 (4)	C93—O93—C931—O931	2.1 (4)
C3—O3—C31—C32	−178.5 (3)	C93—O93—C931—C932	−179.9 (3)
C4—C5—O5—C51	120.3 (3)	C94—C95—O95—C951	−120.5 (3)
C6—C5—O5—C51	−64.9 (4)	C96—C95—O95—C951	65.3 (4)
C5—O5—C51—O51	1.9 (4)	C95—O95—C951—O951	3.1 (4)
C5—O5—C51—C52	−178.6 (3)	C95—O95—C951—C952	−176.2 (3)

### Hydrogen-bond geometry (Å, °)

**Table:**

*D*—H···*A*	*D*—H	H···*A*	*D*···*A*	*D*—H···*A*
C52—H52A···O31^i^	0.96	2.53	3.363 (4)	145
C52—H52C···O11^i^	0.96	2.31	3.242 (4)	163
C932—H93A···O911^ii^	0.96	2.51	3.292 (4)	139
C952—H95C···O951^iii^	0.96	2.54	3.474 (4)	165
C4—H4···Cg2^iv^	0.93	2.68	3.460 (3)	141
C12—H12C···Cg1^v^	0.96	2.86	3.604 (4)	135
C912—H91B···Cg2^vi^	0.96	2.84	3.658 (4)	143
C94—H94···Cg1^vii^	0.93	2.67	3.435 (3)	140

Symmetry codes: (i) *x*+1/2, −*y*+1/2, *z*+1/2; (ii) −*x*+1, −*y*+1, −*z*+1; (iii) −*x*, −*y*+1, −*z*; (iv) −*x*+3/2, *y*−1/2, −*z*+1/2; (v) *x*−1, *y*, *z*; (vi) *x*+1, *y*, *z*; (vii) −*x*+1/2, *y*+1/2, −*z*+1/2.
